# The Utility of Bronchoscopy in Hydralazine-Induced ANCA-Associated Vasculitis

**DOI:** 10.1155/2023/1461011

**Published:** 2023-04-15

**Authors:** Atulya Aman Khosla, Hollie Saunders, Scott Helgeson, Hiroshi Hikida, Nabeel Aslam, Fadi Salem, Sam Albadri, Hassan Baig

**Affiliations:** Mayo Clinic Florida, Jacksonville, FL 32224, USA

## Abstract

Hydralazine is a vasodilator used for the management of hypertension, heart failure, and hypertensive emergencies in pregnancy. It has been implicated in the causation of drug-induced lupus erythematosus (DLE) and rarely with ANCA-associated vasculitis (AAV), which may present as a pulmonary-renal syndrome and be rapidly fatal. Herein, we describe a case of hydralazine-associated AAV presenting as acute kidney injury with the use of early bronchoalveolar lavage (BAL) with serial aliquots to aid with diagnosis. Our case highlights how, in the correct clinical setting, BAL can act as a rapid diagnostic test to help guide quicker treatment to allow for better patient outcomes.

## 1. Introduction

Hydralazine is a direct-acting arteriolar vasodilator used as an adjunctive treatment for hypertension, heart failure with reduced ejection fraction, and hypertensive emergencies during pregnancy [1].

Hydralazine has often been implicated as a causative factor in drug-induced lupus erythematosus (DLE), but it rarely causes antineutrophil cytoplasmic antibody- (ANCA-) associated vasculitis (AAV) [2]. DLE is characterized by fever, polyarthralgia, myalgia, and serositis, in addition to an increase in erythrocyte sedimentation rate (ESR) and antinuclear antibodies (ANA) titer [3]. On the other hand, AAV is associated with leukocytoclastic vasculitis, pulmonary infiltrates, elevated ANCA levels, and pauci-immune glomerulonephritis on renal biopsy [4, 5]. It is theorized that both DLE and AAV might represent a spectrum of the same pathology, and ANCAs may serve as an essential means of differentiating the two before a biopsy [6].

There are several proposed mechanisms of hydralazine-associated AAV [7, 8]. First, hydralazine can bind to myeloperoxidase (MPO), subsequently leading to neutrophil apoptosis and the generation of autoantibodies. Second, hydralazine may induce epigenetic silencing of MPO and proteinase-3 (PR3), which leads to increased expression of neutrophil autoantigens. Hydralazine-induced AAV has been more commonly reported in slow acetylators owing to slower metabolism and subsequently increased chances for a breakdown in tolerance [9].

Hydralazine-induced AAV is a rare phenomenon, with pulmonary-renal syndrome being its most severe presentation [10]. Prompt diagnosis of hydralazine-induced AAV followed by discontinuation of the drug combined with optimal medical management results in improved clinical outcomes [10]. Herein, we present a case of a 72-year-old woman with a history of hypertension on hydralazine who presented with slowly progressive dyspnea and acute kidney injury. In our case, high clinical suspicion and diagnostic testing, including bronchoscopy, allowed for rapid diagnosis and initiation of therapy while awaiting biopsy and serology for confirmation.

## 2. Case

A 72-year-old woman presented to the hospital with symptoms of slowly progressive dyspnea on exertion and fatigue. Before admission, she had required four transfusions of packed red blood cells over the prior two weeks for the management of symptomatic anemia from an unknown cause. The patient had underlying essential hypertension, chronic kidney disease stage 3, and type 2 diabetes mellitus. Active home medications included losartan 100 mg daily, hydralazine 50 mg t.i.d., and carvedilol 6.25 mg b.i.d, with hydralazine added about a year and a half prior to this hospital admission.

On admission, her vital signs were significant for an oxygen saturation of 98% on room air, respiratory rate of 22 breaths per minute, a temperature of 36.6°C, and blood pressure of 195/83 mmHg. Pertinent laboratory testing upon admission demonstrated hemoglobin of 8.2 g/dL, white blood cell count 5.2 × 10^9^/L, potassium 5.3 mmol/L, bicarbonate 15 mmol/L, phosphorous 4.6 mg/dL, blood urea nitrogen 61 mg/dL, and a creatinine of 5.11 mg/dL, which was significantly increased from a baseline of 1.3-1.5 mg/dL measured two weeks prior. Her estimated glomerular filtration rate was <15 mL/min/BSA, with a C-reactive protein of 63.4 mg/L. Urinalysis showed 95 red blood cells/hpf and protein of 100 mg/dL with no RBC casts.

**Figure 1:**
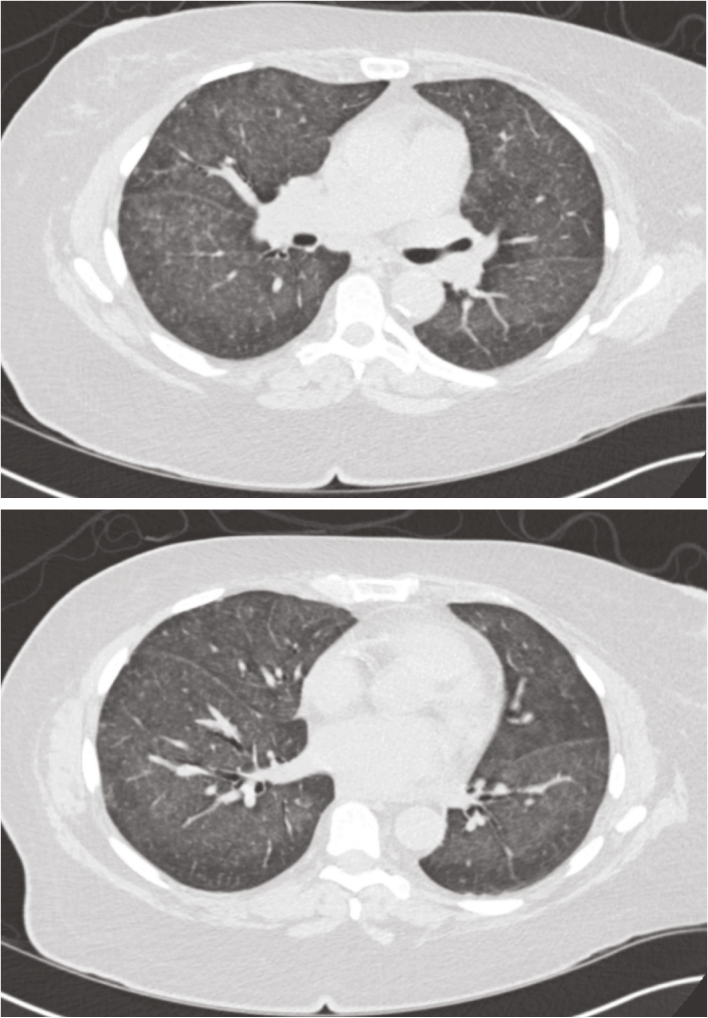
Admission noncontrast chest CT demonstrating diffuse confluent ground-glass opacities in all five lobes with prominent subpleural sparing.

**Figure 2:**
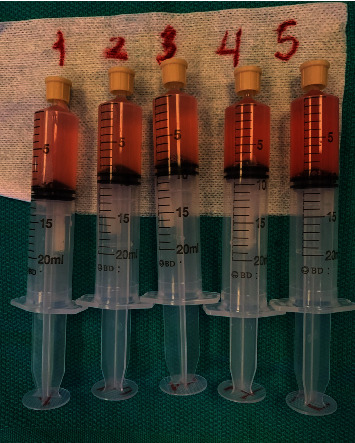
Bronchoalveolar lavage serial aliquots. Aspirate marked 1 correlates with the first aliquot with ascending numbers denoting sequentially taken aliquots. Findings demonstrate progressive blood-tinged fluid consistent with alveolar hemorrhage.

The initial chest X-ray showed diffuse alveolar and interstitial opacities without pleural effusions. Subsequently, a chest computed tomography (CT) was significant for diffuse confluent ground-glass opacities in all five lobes with prominent subpleural sparing (Figure 1). Based on these CT findings, even with the absence of significant pulmonary complaints, the patient underwent bronchoscopy with bronchoalveolar lavage and serial aliquots collected from the right middle lobe. Five syringes of 20 ml of saline were instilled; between each instillation, the fluid was manually aspirated with a new syringe used each time and marked. The aspirated fluid from the lavage was pink-tinged, and a deepening of the pink color was noticed with subsequent aliquots (Figure 2). This finding was consistent with diffuse alveolar hemorrhage. The patient was immediately started on pulsed dose steroids of intravenous methylprednisolone 500 mg daily for three days. Infectious testing from the bronchoalveolar lavage remained negative.

**Figure 3:**
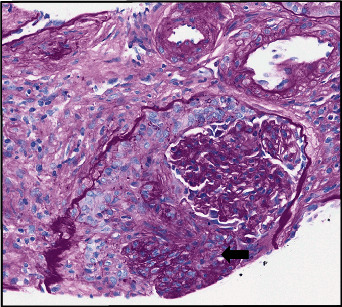
A glomerulus is involved by fibrocellular crescent (black arrow) occupying most of the Bowman space of the glomerulus. The glomerular capillary loops are collapsed and corrugated (Periodic Acid-Schiff, original magnification ×40).

**Figure 4:**
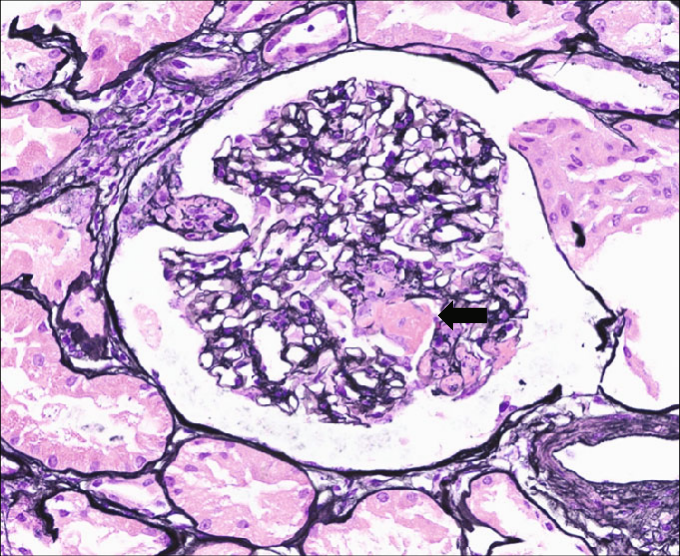
Another glomerulus showing a segmental fibrinoid necrosis (black arrow) causing obliteration and break of capillary loops (Methenamine silver stain, original magnification ×40).

Autoimmune serologies obtained on admission resulted after the bronchoscopy and showed an antinuclear antibody of 2.6 U, dsDNA antibody of 296 IU/mL, myeloperoxidase antibody 3.4 U, proteinase 3 antibody 5.2 U, and a positive perinuclear cytoplasmic neutrophil antibody (p-ANCA). Cytoplasmic-ANCA, antiglomerular basement membrane antibodies, hepatitis serologies, and cryoglobulins were negative, along with normal complement C3 and C4 levels. The patient subsequently underwent a renal biopsy which exhibited a picture of pauci-immune necrotizing crescentic glomerulonephritis (Figures 3 and 4).

Based on her clinical, laboratory, and pathology findings, she was diagnosed with hydralazine-induced AAV. After completing pulse steroids, the patient was started on high-dose oral prednisone and rituximab. Hydralazine was discontinued and flagged in her medical record as an allergy.

## 3. Discussion

ANCA-associated vasculitis is a well-described clinical entity and has an incidence of about 10–20 cases per million [11]. The extent of the incidence of drug-induced AAV is largely unknown; however, this association was supported after the discovery of ANCAs and their target antigens PR3 and MPO [7]. Reports of this type of vasculitis being associated with the use of specific drugs have been published, and improvement with the discontinuation of those agents supports the diagnosis [9]. The early case series of patients who were ANCA positive and exposed to medications such as hydralazine, allopurinol, and propylthiouracil leading to vasculitis emerged in the 1980s [12, 13]. The incidence of hydralazine-induced AAV varies from 5.4% in patients on a dose of 100 mg/day to 10.4% with a dose of 200 mg/day for >3-year duration [9].

Risk factors that have been shown to predispose to the occurrence of hydralazine-induced AAV include using a cumulative dose of more than 100 g, female gender, and underlying thyroid disease [14]. In addition, the presence of the HLA-DR4 genotype, slow hepatic acetylation, and the null gene for C4 are also associated with an increased incidence of the disease [14, 15].

Findings in our case are similar to other previously described reports, which include the presence of a significant titer of ANA and ANCA antibodies [6, 7, 10, 16]. In addition, the presence of pauci-immune glomerulonephritis on biopsy is commonly reported in cases of hydralazine-induced vasculitis [17, 18]. The most common theme for good outcomes is the need for prompt diagnosis and treatment, most especially the prevention of respiratory failure and the salvage of renal function. With regard to renal outcomes, Suneja et al. reported full renal recovery in a case series of 4 patients experiencing hydralazine-induced glomerulonephritis with appropriate treatment [17]. Timlin et al. demonstrated progression to end-stage renal disease and/or multiorgan involvement despite immunosuppressive therapy in patients with hydralazine-associated vasculitis and emphasized early diagnosis of the pathology [19]. Across the previously described cases, the diagnosis was made based on the presence of specific antibodies and/or renal biopsy results. Delays can be experienced while waiting for the specific serologies to result and for a renal biopsy to be arranged and analyzed. Aeddula et al. reported initiation of therapy only after the arrival of the renal biopsy result [7]. The case was complicated by acute respiratory failure and the need for initiation of renal replacement therapy [7]. Similarly, Padala et al. reported treatment initiation only after the availability of serology results, and their patient required dialysis [6]. Moreover, despite mentioning bronchoscopy findings with blood-lined bronchi, Al-Abdouh et al. did not use BAL with serial aliquots in order to diagnose pulmonary hemorrhage. Treatment was initiated after the receipt of the renal biopsy result, and the patient required dialysis [10].

As evidenced by this reported case, there are indicators within the history and early diagnostic testing which can be used to raise clinical suspicion and even to potentially initiate therapy. Testing which can increase suspicion and may be rapidly obtained includes spun urine microscopy with direct visualization of red blood cell casts and bronchoscopy with serial aliquots indicative of diffuse alveolar hemorrhage [20]. In our case, spun urine microscopy did not reveal any red blood cell casts, but due to suggestive chest CT findings, even in the absence of significant pulmonary symptoms, we were able to perform a bronchoscopy within 24 hours of admission with findings consistent with diffuse alveolar hemorrhage. Based on this result, we were able to initiate therapy before serological and biopsy results. In our case, the patient did not require dialysis and remained without any oxygen requirement throughout the hospitalization.

What distinguishes our case from previously reported cases of hydralazine-induced AAV is the utilization of bronchoscopy and BAL as a rapid diagnostic test, indicating that CT and BAL findings could result in the initiation of earlier therapy. Bronchoscopy is a relatively low-risk procedure, with complication rates ranging from 0.5 to 1% [21, 22]. A concern would be worsening hypoxia in patients already with hypoxic respiratory failure, so careful patient selection would be required.

## 4. Conclusion

Timely diagnosis is required in the case of hydralazine-induced AAV to ensure drug cessation and initiation of therapy. In addition to high clinical suspicion and suggestive history findings, procedures such as urine microscopy of the centrifuged specimen, chest CT, and bronchoscopy can further raise suspicion and result in more timely therapy.

## Data Availability

The data used to support the findings of this study are available from the corresponding author upon request.
